# DNACPR: Utilising Poster Interventions to Improve Compliance With Scottish Government Guidance

**DOI:** 10.1192/bjo.2024.431

**Published:** 2024-08-01

**Authors:** Aized Raza Shahbaz, James Herron

**Affiliations:** 1 Gartnavel Royal Hospital, Glasgow, United Kingdom; 2 Leverndale Hospital, Glasgow, United Kingdom

## Abstract

**Aims:**

- Study current practices in Old Age Psychiatry (OAP) wards regarding Do Not Attempt Cardiopulmonary Resuscitation (DNACPR) documentation.
- Create an intervention to improve compliance with Scottish Government guidance.

Hypothesis:

- An intervention could improve likely inconsistencies in current DNACPR practices.

Background: DNACPR forms are a contentious issue in the media, impacting patients and families’ views. The Scottish Government's ‘Cardiopulmonary resuscitation decisions – integrated adult policy: guidance’ from 2016 seeks to prevent inappropriate attempts at CPR and subsequent distress to patients and families. It makes various recommendations for clinicians when making these decisions and completing DNACPR forms.

**Methods:**

This was a two-cycle retrospective audit utilising physical and electronic notes for all patients across two OAP wards at the Vale of Leven Hospital, Alexandria. Data were collected on demographics, presence and adequacy of DNACPR forms based on Scottish Government guidance. Between the first (12/09/22) and second (25/11/22) cycle, a poster to aid DNACPR decisions and documentation was created and displayed in the ward office.

**Results:**

There were a total of 13 patients in cycle 1 and 14 patients in cycle 2. The number of patients with forms increased from 3 to 8 between cycles (including all those with organic diagnoses in cycle 2). Between cycle 1 and 2, there were improvements in the proportion of forms: completed at admission (66.7% to 87.5%, respectively), correctly filed (66.7% to 100%), with review timeframes specified (0% to 62.5%) and consultant signatures (33.3% to 100%). The mean age of patients with DNACPR forms was higher than those without forms in both cycles (86.7 and 85.7 in cycle 1 respectively versus 77.9 and 77.7 in cycle 2). The mean number of comorbidities did not vary significantly between those with and without forms or between cycles.

**Conclusion:**

The project revealed various shortcomings in DNACPR practices across both wards. The creation of a poster intervention helped to improve DNACPR practices and compliance with Scottish Government guidance. Despite this, notable areas for improvement still remain. Incorporating these new practices into hospital policy alongside more audit cycles could aid further progress in outstanding areas for improvement.

